# Predictive value of 3′-deoxy-3′-^18^F-fluorothymidine PET in the early response to anti-programmed death-1 therapy in patients with advanced non-small cell lung cancer

**DOI:** 10.1136/jitc-2021-003079

**Published:** 2021-07-21

**Authors:** Masayuki Sato, Yukihiro Umeda, Tetsuya Tsujikawa, Tetsuya Mori, Miwa Morikawa, Masaki Anzai, Yuko Waseda, Maiko Kadowaki, Yasushi Kiyono, Hidehiko Okazawa, Tamotsu Ishizuka

**Affiliations:** 1 Third Department of Internal Medicine, University of Fukui, Eiheiji, Fukui, Japan; 2 Department of Internal Medicine, Municipal Tsuruga Hospital, Tsuruga-shi, Fukui, Japan; 3 Biomedical Imaging Research Center, University of Fukui, Eiheiji, Fukui, Japan; 4 Department of Internal Medicine, Tokyo Shinagawa Hospital, Tokyo, Japan

**Keywords:** immunotherapy, lung neoplasms, tumor biomarkers

## Abstract

**Background:**

Anti-programmed death-1 (anti-PD-1) therapy has shown clinical success in patients with advanced non-small cell lung cancer (NSCLC). However, it is difficult to evaluate the early response to anti-PD-1 therapy. We determined whether changes in 3′-deoxy-3′-[^18^F]-fluorothymidine (^18^F-FLT) PET parameters before and soon after treatment initiation predicted the therapeutic effect of anti-PD-1 antibody.

**Methods:**

Twenty-six patients with advanced NSCLC treated with anti-PD-1 antibody were enrolled prospectively and underwent ^18^F-FLT PET before and at 2 and 6 weeks after treatment initiation. Changes in maximal standardized uptake value (ΔSUV_max_), proliferative tumor volume (ΔPTV) and total lesion proliferation (ΔTLP) of the lesions were calculated and evaluated for their associations with the clinical response to therapy.

**Results:**

The disease control rate was 64%. Patients with non-progressive disease (non-PD) had significantly decreased TLP at 2 weeks, and decreased SUV_max_, PTV, and TLP at 6 weeks, compared with those with PD, while three of eight (37.5%) patients who responded had increased TLP from baseline at 2 weeks (ie, pseudoprogression). Among the parameters that changed between baseline and 2 weeks, ΔPTV0-2 and ΔTLP0-2 had the highest accuracy (76.0%) to predict PD. Among the parameters that changed between baseline and 6 weeks, ΔSUV_max_0-6, ΔPTV0-6 and ΔTLP0-6 had the highest accuracy (90.9%) to predict PD. ΔTLP0-2 (≥60%, HR 3.41, 95% CI 1.34–8.65, p=0.010) and ΔTLP0-6 (≥50%, HR 31.4, 95% CI 3.55 to 276.7, p=0.0019) were indicators of shorter progression-free survival.

**Conclusions:**

Changes in ^18^F-FLT PET parameters may have value as an early predictive biomarker for the response to anti-PD-1 therapy in patients with NSCLC. However, it should be noted that pseudoprogression was observed in ^18^F-FLT PET imaging at 2 weeks after treatment initiation.

**Trial registration number:**

jRCTs051180147.

## Introduction

Immune checkpoint inhibitors including anti-programmed death-1 (anti-PD-1) antibody play a central role in the treatment of advanced non-small cell lung cancer (NSCLC). Although a significant number of patients with cancer benefit from anti-PD-1 therapy, many fail to have clinical responses.^1^ To date, the most successful biomarker associated with response to anti-PD-1 therapy is tumor cell expression of programmed death-ligand 1 (PD-L1).^2 3^ However, more than half of NSCLC patients with high PD-L1 expression (≥50%) do not respond to anti-PD-1 therapy, while 9%–17% of those without PD-L1 expression (<1%) do respond.^4–6^

The therapeutic response to immunotherapy is generally assessed by morphological changes using CT. However, it is difficult to assess the response to immunotherapy at an early phase using CT because of two immunotherapy-specific responses. First, a phenomenon known as pseudoprogression has been described in patients with various tumors who receive immunotherapy and experience an objective response after initial tumor progression.^7 8^ Second, a paradoxical acceleration in tumor growth early after immunotherapy initiation known as hyperprogression,^9^ which is correlated with worse prognosis, has been reported. Therefore, when tumors grow early after anti-PD-1 therapy initiation, it is difficult to determine whether the growth is true or not. For these reasons, some radiological criteria for immunotherapy have been recently developed to assess tumor responses correctly.^10–12^ According to these criteria, a confirmatory CT at 4–8 weeks after the first findings of progression by CT is required to distinguish progressive disease (PD) from pseudoprogression. Thus, it is impossible to predict the early response to immunotherapy using CT. Therefore, there is an urgent need to establish more accurate biomarkers for the response to immunotherapy, which can help to curtail ineffective and potentially toxic therapies.

[^18^F ]-Fluoro-2-deoxy-D-glucose (^18^F-FDG) positron emission tomography (PET) has recently been applied to assess the response to chemotherapy in various tumor types.^13^ One important problem in the use of ^18^F-FDG PET to evaluate the response to immunotherapy is ^18^F-FDG accumulation in activated glucose-consuming inflammatory cells in and around tumors during immunotherapy. In fact, it has recently been reported that resected lung cancer lesions with major pathological responses to anti-PD-1 neoadjuvant therapy show increased CD8+ T cell and macrophage infiltration into the tumor.^14^ As a result, in our previous study, 33.3% of responders showed an increase in ^18^F-FDG uptake 2 weeks after anti-PD-1 therapy.^15^ Thus, there is an increasing clinical need to use other PET tracers to avoid this disadvantage.

3′-Deoxy-3′-[^18^F ]-fluorothymidine (^18^F-FLT) PET has been proposed as an imaging method that can evaluate cell proliferation indirectly,^16^ since a significant correlation has been found between ^18^F-FLT uptake and Ki-67 in various tumors.^17^ ^18^F-FLT PET has been described as superior to ^18^F-FDG PET for the quantitative assessment of tumor cell proliferation.^18^ Recently, ^18^F-FLT PET imaging has been shown to be useful in predicting treatment response to chemotherapy, chemoradiotherapy, and molecularly targeted drugs.^19 20^ However, to date, there is no report applying ^18^F-FLT PET imaging to evaluate early responses to immunotherapy for NSCLC. The aim of this study was to evaluate whether changes in tumor ^18^F-FLT accumulation before and after the initiation of anti-PD-1 therapy could accurately predict tumor response as assessed by immune-related Response Evaluation Criteria in Solid Tumor (irRECIST) criteria^21^ and patient survival.

## Materials and methods

### Study design and patient selection

This study protocol was performed in accordance with the Declaration of Helsinki and the guidelines for Good Clinical Practice. The clinical trial was registered at https://jrct.niph.go.jp/. This study was conducted prospectively in patients with advanced NSCLC who were treated at the University of Fukui Hospital from June 2017 to July 2019, and the protocol-defined final analysis was performed on January 26, 2020. Eligibility criteria included histologically/cytologically confirmed advanced or recurrent NSCLC measurable by irRECIST, candidates for anti-PD-1 antibody (nivolumab or pembrolizumab) therapy, and an Eastern Cooperative Oncology Group performance status (ECOG-PS) of 0–2. Exclusion criteria included a metallic device in the body, claustrophobia, and pregnant woman.

For the evaluation of tumor expression of PD-L1, immunohistochemical staining using 22C3 pharmDx assay (Agilent, Santa Clara, California, USA) and a Dako Autostainer Link 48 platform (Dako, Carpenteria, California, USA) was performed by LSI Medience Corporation (Tokyo, Japan) as previously described.^15^ The PD-L1 tumor proportion score (TPS) was assessed by two trained tissue technicians and one trained pathologist hired by a vendor (LSI Medience Corporation).

Patients were treated with nivolumab or pembrolizumab at the discretion of each attending physician. Pembrolizumab was given as an intravenous 200 mg fixed dose every 3 weeks, and nivolumab was given as an intravenous either 3 mg/kg body weight or 240 mg fixed dose every 2 weeks because of the change of dosage and administration of nivolumab during the study period. Treatment was administrated until disease progression or unacceptable toxicity. Baseline ^18^F-FLT PET and CT were performed prior to treatment initiation (within 7 days), and the subsequent PET and CT evaluations were performed at 2 weeks (±3 days) and 6 weeks (±5 days) after treatment initiation. Tumor response was assessed using irRECIST at weeks 2 and 6 and at least every 8 weeks thereafter.^22^ Based on the best overall response as assessed by irRECIST, the patients were dichotomized into those with PD and non-PD (complete response (CR), partial response (PR), or stable disease (SD)) to evaluate the association between changes in ^18^F-FLT uptake and tumor response.

### ^18^F-FLT PET image acquisition

In this study, ^18^F-FLT was radiosynthesized in a TRACERlab MX-FDG (GE Healthcare) using an FLT kit (ABX GmbH) as previously described.^15^ No-carrier-added ^18^F-fluoride was produced via the ^18^O(p, n)^18^F reaction from >98% enriched ^18^O-water (Cambridge Isotope Laboratories) on an RDS eclipse RD/HP medical cyclotron (Siemens Healthcare). The radiochemical purity of the final product was >99%.

All patients were scanned on a whole-body simultaneous 3.0T PET/MR scanner (Signa PET/MR, GE Healthcare, Waukesha, Wisconsin, USA) as previously described.^15 23^ PET scans were obtained at 50 min after the intravenous injection of 185 MBq of ^18^F-FLT. Anatomic coverage was from the thorax to the pelvis. PET data were acquired with a 10 min/bed position (89 slices/bed) in three beds with a 24-slice overlap. For MR-based attenuation correction to recognize body tissues as soft tissue, fat, and air, a two-point Dixon 3D volumetric interpolated T1-weighted fast SPGR sequence (repetition time/echo time (TE)1/TE2: 4.0/1.1/2.2 ms; field of view: 50×37.5 cm; matrix: 256×128; slice thickness/overlap: 5.2/2.6 mm; 120 image/slab; imaging time: 18 s) was acquired.

PET images were reconstructed with ordered subset expectation maximization algorithm selecting three iterations and 32 subsets. For the semiquantitative analysis, the reconstructed PET images were converted to standardized uptake value (SUV) images corrected by the injection dose of ^18^F-FLT and subject’s body weight.

### PET image analysis

The ^18^F-FLT PET images were reviewed using a specific software package (RAVAT; Nihon Medi-Physics, Tokyo, Japan). We referred to the methodology of a prior study for quantification of ^18^F-FLT using PET/CT.^24^ For the semiquantitative analysis, three-dimensional volumes of interest were placed on the primary and metastatic tumors, with the exception of bone and liver metastases due to the high background of ^18^F-FLT uptake in the bone marrow and liver. After having examined various SUV thresholds in increments of 0.5, we adopted an SUV of 2.0 as the threshold to define the tumor contour. Since the tumor uptake of ^18^F-FLT is lower than that of ^18^F-FDG, a threshold higher than 2.0 would underestimate the tumor volume, while a threshold lower than 2.0 would include normal soft tissues such as the mediastinum. Therefore, we adopted 2.0 as the most suitable threshold for the volumetric analysis of ^18^F-FLT PET in patients with NSCLC. The maximal SUV (SUV_max_) and the average SUV (SUV_mean_) within the extracted tumor area were calculated, and the extracted tumor volume was defined as the proliferative tumor volume (PTV). Then, the total lesion proliferation (TLP) was calculated according to the formula: TLP=SUV_mean_ × PTV. In patients with multiple tumors, the highest SUV_max_ among lesions was selected for the patient SUV_max_. To assess tumor burden, the sum of PTV and TLP for all measurable lesions was calculated.

For response assessment, the changes of three extracted parameters (SUV_max_, sum of PTV, and sum of TLP) between baseline and at 2 and 6 weeks were calculated as the percentage change (ΔSUV_max_0-2, ΔPTV0-2, ΔTLP0-2, ΔSUV_max_0-6, ΔPTV0-6, and ΔTLP0-6).

### Statistical analysis

The primary endpoint was the predictive value of serial ^18^F-FLT PET findings, including SUV_max_, PTV, and TLP, for tumor response to anti-PD-1 therapy. The secondary endpoints were the predictive value of those parameters for progression-free survival (PFS) and overall survival (OS), defined as the time from initial anti-PD-1 therapy to disease progression or death.

For analysis of individual group differences, we used Mann-Whitney U test. Receiver operating characteristic (ROC) analysis was used to compare the diagnostic capability among serial ^18^F-FLT PET parameters and their optimal thresholds. PFS and OS were evaluated using the Kaplan-Meier method with a log-rank test. Univariate Cox regression analyses were used to determine factors affecting the PFS and OS. All analyses were performed using SPSS Statistics V.22.0 (IBM, Armonk, New York, USA). P values less than 0.05 were considered statistically significant.

## Results

### Patient characteristics

**Figure 1:**
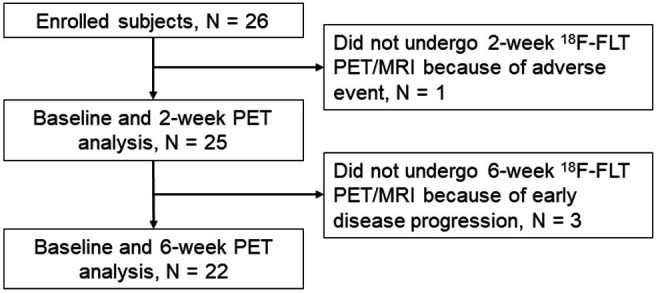
Flow diagram. ^18^F-FLT, 3′-Deoxy-3′-[^18^F ]-fluorothymidine; PET, positron emission tomography.

Twenty-six consecutive patients with advanced NSCLC were prospectively enrolled in this study (figure 1). Of these, one patient did not undergo 2-week ^18^F-FLT PET/MRI because of discontinuance of pembrolizumab treatment due to drug-induced pneumonia, and three patients did not undergo 6-week ^18^F-FLT PET/MRI because of early disease progression. Therefore, 25 patients were analyzed at baseline and at 2-week ^18^F-FLT PET, and 22 patients were analyzed at baseline and at 6-week ^18^F-FLT PET. No adverse events by ^18^F-FLT PET/MRI were observed. Patient characteristics are summarized in table 1. The median age was 70.4 years (range 54–82). The histology of the patients was as follows: 11 (44%) with squamous cell carcinoma, 9 (36%) with adenocarcinoma, 3 (12%) with NSCLC not otherwise specified, 1 (4%) with large cell carcinoma, and 1 (4%) with pleomorphic carcinoma. Twelve (48%) had tumor PD-L1 expression of at least 50%, whereas 10 (40%) had a TPS of <1%.

**Table 1:** Patient characteristics (n=25)

	N (%)
Age, median (range)	70.4 (54–82)
Sex, male/female	23/2 (92/8)
Smoking history	
Current smoker	6 (24)
Former smoker	18 (72)
Never smoked	1 (4)
ECOG-PS	
0	8 (32)
1	14 (56)
2	3 (12)
Histology	
Adenocarcinoma	9 (36)
Squamous cell carcinoma	11 (44)
Large cell carcinoma	3 (12)
Pleomorphic carcinoma	1 (4)
Not otherwise specified	1 (4)
Stage	
III	11 (44)
IV	12 (48)
Recurrence	2 (8)
Driver mutation	
*EGFR*	1 (4)
*ALK*	0 (0)
None	24 (96)
Number of previous regimens	
0	7 (28)
1	11 (44)
2	4 (16)
≥3	3 (12)
PD-L1 expression, tumor proportion score	
<1%	10 (40)
1%–49%	3 (12)
≥50%	12 (48)
Regimen, nivolumab/pembrolizumab	10/15 (40/60)
Confirmed response	
CR	1 (4)
PR	7 (28)
SD	8 (32)
PD	9 (36)

*ALK*, anaplastic lymphoma kinase; CR, complete response; ECOG-PS, Eastern Cooperative Oncology Group performance status; EGFR, epidermal growth factor receptor; PD, progressive disease; PR, partial response; SD, stable disease.

### ^18^F-FLT PET imaging parameters and response assessment

The objective response rate and disease control rate after anti-PD-1 therapy were 32% and 64%, respectively (CR: n=1, PR: n=7, SD: n=8, and PD: n=9) (table 1).

**Figure 2:**
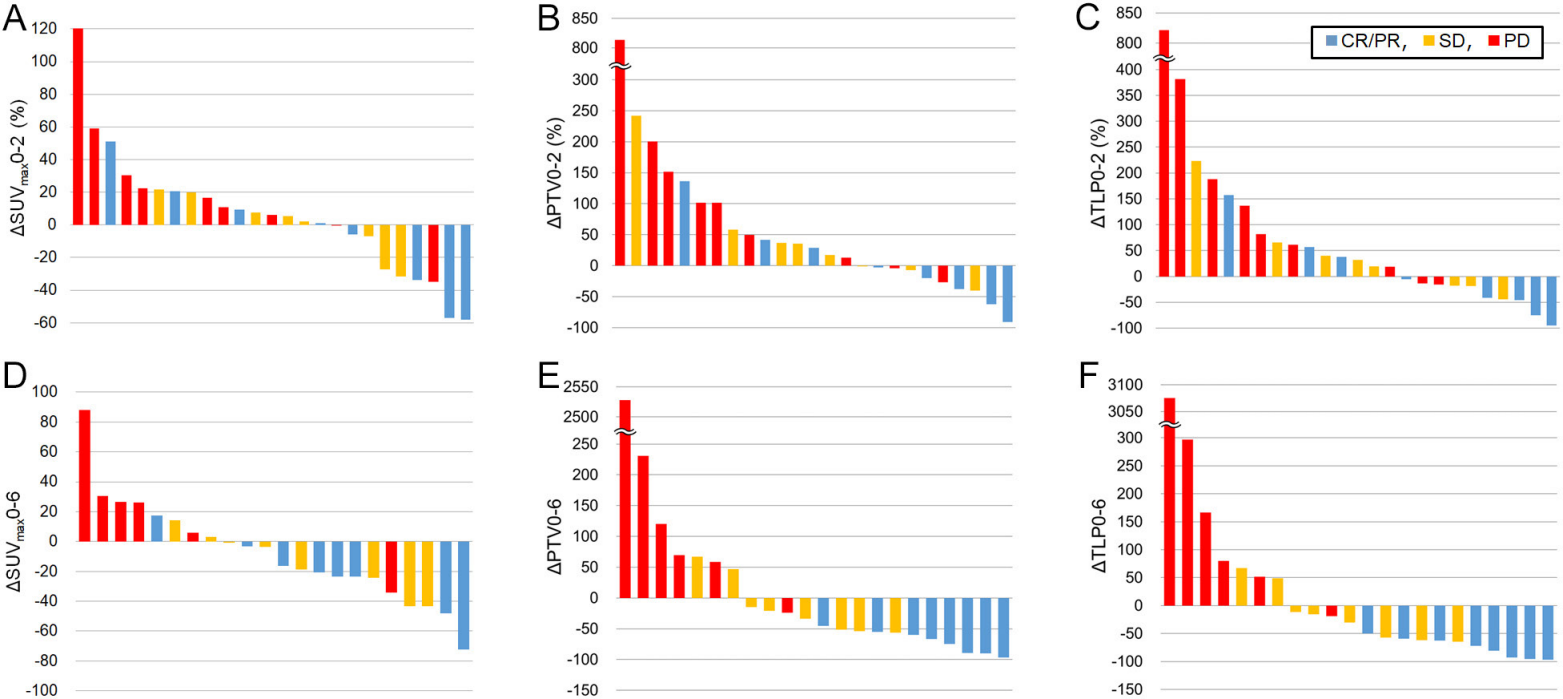
Changes from baseline in ^18^F-FLT PET parameters in anti-PD-1 antibody-treated patients according to treatment response. Per cent changes are shown for ΔSUV_max_0-2 (A), ΔPTV0-2 (B), ΔTLP0-2 (C), ΔSUV_max_0-6 (D), ΔPTV0-6 (E), and ΔTLP0-6 (F) in all patients. Blue, yellow, and red bars show CR/PR, SD, and PD as confirmed by irRECIST, respectively. ΔSUV_max_, changes in maximal standardized uptake value; ^18^F-FLT, 3′-deoxy-3′-[^18^F]-fluorothymidine; CR, complete response; irRECIST, immune-related Response Evaluation Criteria in Solid Tumor; PET, positron emission tomography; PR, partial response; PTV, proliferative tumor volume; SD, stable disease; TLP, total lesion proliferation.

Changes in ^18^F-FLT PET parameters between baseline and 2 and 6 weeks after anti-PD-1 therapy are shown in figure 2 and table 2. Patients with non-PD (CR, PR, and SD) had significantly lower ΔTLP0-2, ΔSUV_max_0-6, ΔPTV0-6, and ΔTLP0-6 than patients with PD, whereas there were no significant differences between these groups in ΔSUV_max_0-2 and ΔPTV0-2. One of eight patients who were responders had increased SUV_max_ from baseline to 6 weeks (ΔSUV_max_0-6 17.3%, figure 2D), while all patients who were responders showed decreased PTV and TLP from baseline to 6 weeks (figure 2E, F).

**Table 2:** Changes from baseline in CT and ^18^F-FLT PET parameters according to confirmed treatment response

	All subjects, n=25	Non-progressive disease, n=16	Progressive disease, n=9	P value*
Changes between baseline and week 2				
ΔSUV_max_0-2, %	6.0 (−58.3 to 120.2)	2.1 (−58.3 to 50.9)	16.8 (−34.8 to 120.2)	0.075
ΔPTV0-2, %	29.0 (−90.7 to 813.2)	12.5 (−90.7 to 241.9)	101.3 (−27.0 to 813.2)	0.066
ΔTLP0-2, %	31.9 (−94.8 to 821.3)	12.7 (−94.8 to 223.2)	81.6 (−15.9 to 821.3)	0.045

	All subjects, n=22	Non-progressive disease, n=16	Progressive disease, n=6	P value*
Changes between baseline and week 6				
ΔSUV_max_0-6, %	−3.4 (-72.3 to 88.1)	−17.5 (-72.3 to 17.3)	26.3 (-34.8 to 88.1)	0.020
ΔPTV0-6, %	−31.2 (−96.9 to 2528.1)	−54.7 (−96.9 to 67.1)	95.0 (−23.9 to 2528.2)	0.0017
ΔTLP0-6, %	−24.9 (−97.1 to 3075.2)	−58.1 (−97.1 to 66.8)	123.2 (−19.2 to 3075.2)	0.0022

*P values compare the non-progressive and progressive disease groups.

^18^F-FLT, 3′-deoxy-3′-[^18^F]-fluorothymidine; PET, positron emission tomography; PTV, proliferative tumor volume; SUV, standardized uptake value; TLP, total lesion proliferation.

ROC curves were constructed to determine the appropriate cut-off values for dichotomization of patients according to parameters of PET imaging (online supplemental figure 1). According to the ROC curves, the appropriate cut-off values for ΔSUV_max_0-2, ΔPTV0-2, ΔTLP0-2, ΔSUV_max_0-6, ΔPTV0-6, and ΔTLP0-6 were 10%, 45%, 60%, 5%, 50%, and 50%, respectively. Table 3 presents the predictive value of these parameters for distinguishing PD and non-PD based on the Δdiameter according to irRECIST (cut-off 20%), ΔSUV_max_ according to Positron Emission Tomography Response Criteria in Solid Tumors (PERCIST) (cut-off 30%)^25^ and the ROC-determined cut-off values. Among the parameters of the changes between baseline and week 2, ΔPTV0-2 (cut-off 45%), and ΔTLP0-2 (cut-off 60%) had the highest accuracy (76.0%). Six of 22 patients who underwent 6-week ^18^F-FLT PET scans experiend PD. Among the parameters of the changes between baseline and week 6, ΔSUV_max_0-6 (cut-off 20%), ΔPTV0-6 (cut-off 50%), and ΔTLP0-6 (cut-off 50%) had the highest accuracy (90.9%) to predict PD. The median time to progression of the nine PD patients as assessed by irRECIST was 41 days (range 12–99 days).

10.1136/jitc-2021-003079.supp1 Supplementary data

**Table 3:** Power of serial CT and PET/MRI parameters to predict progressive disease

	Cut-off (%)	Sensitivity % (N)	Specificity % (N)	PPV% (N)	NPV% (N)	Accuracy % (N)
Changes between baseline and week 2						
Δdiameter0-2	20	11.1 (1/9)	100 (16/16)	100 (1/1)	66.7 (16/24)	68.0 (17/25)
ΔSUV_max_0-2	30	33.3 (3/9)	93.8 (15/16)	75.0 (3/4)	71.4 (15/21)	72.0 (18/25)
ΔPTV0-2	30	66.7 (6/9)	62.5 (10/16)	50.0 (6/12)	76.9 (10/13)	64.0 (16/25)
ΔTLP0-2	30	66.7 (6/9)	56.3 (9/16)	46.2 (6/13)	75 (9/12)	60.0 (15/25)
ΔSUV_max_0-2	10	66.7 (6/9)	75.0 (12/16)	60.0 (6/10)	80 (12/15)	72.0 (18/25)
ΔPTV0-2	45	66.7 (6/9)	81.3 (13/16)	66.7 (6/9)	81.3 (13/16)	76.0 (19/25)
ΔTLP0-2	60	66.7 (6/9)	81.3 (13/16)	66.7 (6/9)	81.3 (13/16)	76.0 (19/25)
Changes between baseline and week 6						
Δdiameter0-6	20	50.0 (3/6)	100 (16/16)	100 (3/3)	84.2 (16/19)	86.4 (19/22)
ΔSUV_max_0-6	30	33.3 (2/6)	100 (16/16)	100 (2/2)	80.0 (16/20)	81.8 (18/22)
ΔPTV0-6	30	83.3 (5/6)	87.5 (14/16)	71.4 (5/7)	93.3 (14/15)	86.4 (19/22)
ΔTLP0-6	30	83.3 (5/6)	87.5 (14/16)	71.4 (5/7)	83.3 (14/15)	86.4 (19/22)
ΔSUV_max_0-6	20	66.7 (4/6)	100 (16/16)	100 (4/4)	88.9 (16/18)	90.9 (20/22)
ΔPTV0-6	50	83.3 (5/6)	93.8 (15/16)	83.3 (5/6)	93.8 (15/16)	90.9 (20/22)
ΔTLP0-6	50	83.3 (5/6)	93.8 (15/16)	83.3 (5/6)	93.8 (15/16)	90.9 (20/22)

NPV, negative predictive value; PET, positron emission tomography; PPV, positive predictive value; PTV, proliferative tumor volume; SUV, standardized uptake value; TLP, total lesion proliferation.

### Association between changes in ^18^F-FLT PET parameters and PFS and prognosis

**Figure 3:**
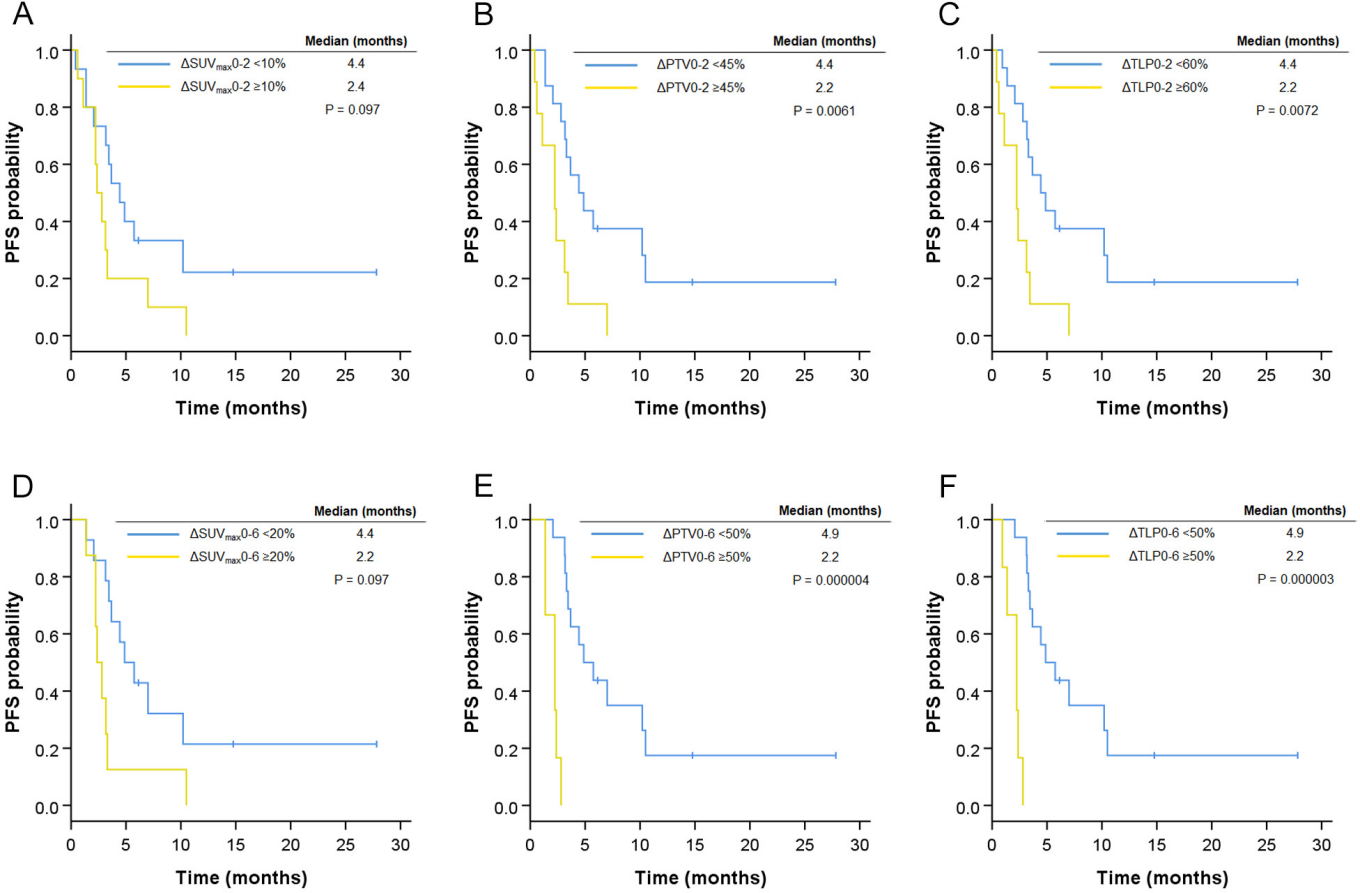
Progression-free survival (PFS) of anti-PD-1 antibody-treated patients stratified by changes in ^18^F-FLT PET parameters. Kaplan–Meier curves of PFS stratified by changes between baseline and week 2 (n=25, ΔSUV_max_0-2 (A), ΔPTV0-2 (B), ΔTLP0-2 (C)), and changes between baseline and week 6 (n=22, ΔSUV_max_0-6 (D), ΔPTV0-6 (E), ΔTLP0-6 (F)) with cut-off values as determined by ROC curve analysis. ^18^F-FLT, 3′-deoxy-3′-[^18^F ]-fluorothymidine; PET, positron emission tomography; PTV, proliferative tumor volume; ROC, receiver operating characteristic; SUV, standardized uptake value; TLP, total lesion proliferation.

The median follow-up period of PFS for censored cases was 10.5 months (range 6.1–27.8 months). Twenty-two patients showed disease progression, and 11 died from lung cancer during the study period. The median PFS of all patients was 3.2 months. Kaplan-Meier curves for PFS stratified by the imaging cut-off values are shown in figure 3. Significant differences in PFS were observed between the groups stratified by ΔPTV0-2, ΔTLP0-2, ΔSUV_max_0-6, ΔPTV0-6, and ΔTLP0-2. In contrast, no significant difference was found between changes in ^18^F-FLT PET parameters and overall survival in this study (data not shown).

A univariate Cox model was used to evaluate predictors of PFS and OS (table 4). We found that ΔTLP0-2 (≥60, HR 3.41, 95% CI 1.34 to 8.65, p=0.010) and ΔTLP0-6 (≥50%, HR 31.4, 95% CI 3.55 to 276.7, p=0.0019) were indicators of shorter PFS, whereas age, ECOG-PS, histology, and TPS were not significant.

**Table 4:** Prognostic factors for progression-free survival (PFS) and overall survival (OS) using Cox models

	PFS			OS		
	HR	95% CI	P value	HR	95% CI	P value
Age, ≥70 years	1.02	0.43 to 2.41	0.97	1.70	0.50 to 5.81	0.40
ECOG-PS, 1–2	2.21	0.77 to 6.38	0.14	1.32	0.34 to 5.05	0.68
Number of previous regimens, ≥2	1.11	0.43 to 2.88	0.83	1.34	0.34 to 5.17	0.68
Stage, IV or recurrence	1.49	0.60 to 3.69	0.39	1.59	0.47 to 5.45	0.46
Histology, squamous cell carcinoma	1.67	0.70 to 3.98	0.25	1.44	0.42 to 5.00	0.56
TPS, <50%	1.52	0.63 to 3.65	0.35	0.79	0.23 to 2.74	0.71
ΔTLP0−2, ≥60%	3.41	1.34 to 8.65	0.010	2.05	0.59 to 7.11	0.26
ΔTLP0−6, ≥50%*	31.4	3.55 to 276.7	0.0019	2.14	0.48 to 9.63	0.32

*N=22. Three patients did not undergo ^18^F-FLT PET at 6 weeks after treatment initiation because of early disease progression.

ECOG-PS, Eastern Cooperative Oncology Group performance status; TLP, total lesion proliferation; TPS, tumor proportion score.

### Response patterns of ^18^F-FLT accumulation to immunotherapy

**Figure 4:**
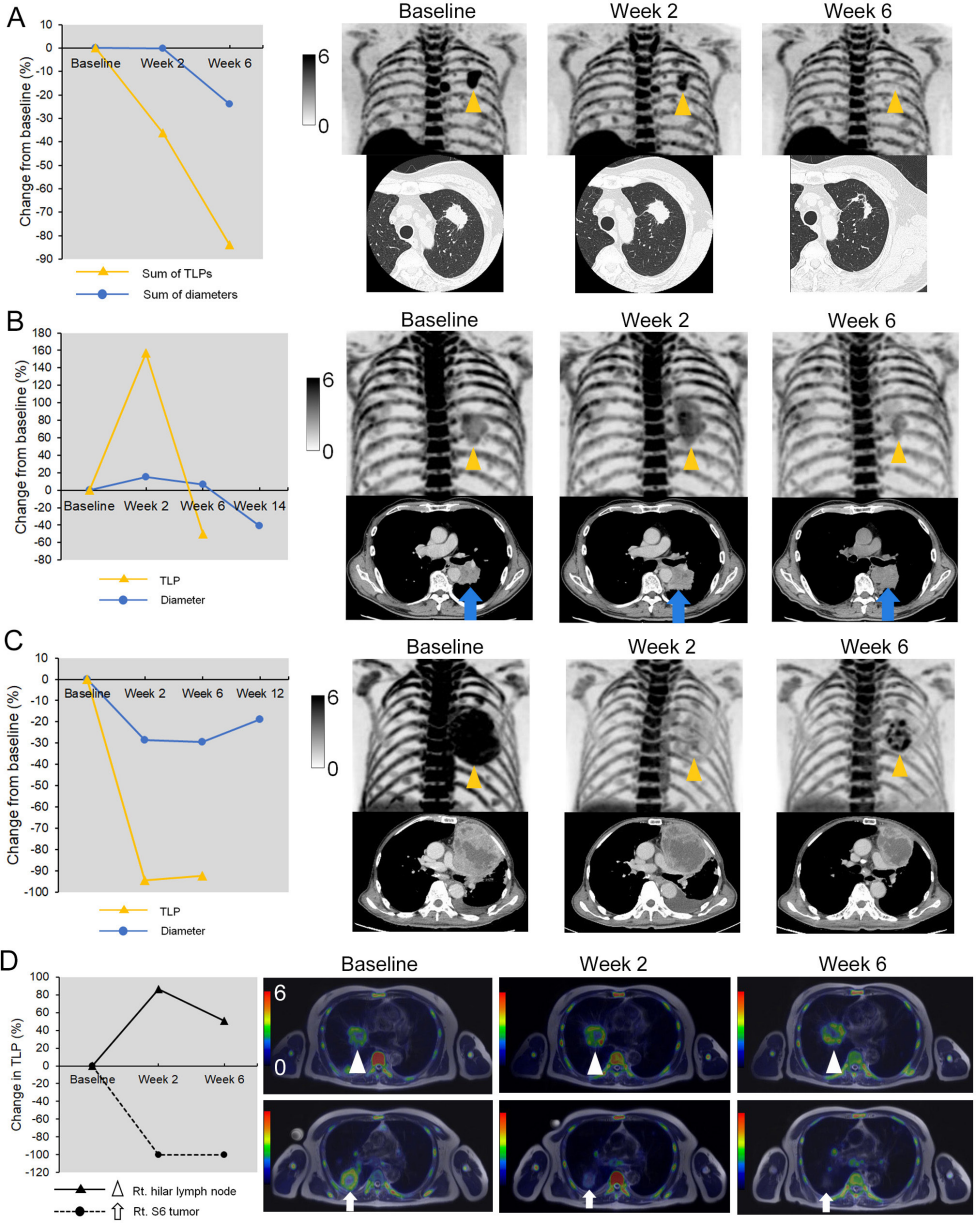
Representative images with various response patterns by ^18^F-FLT PET. (A) A 65-year-old woman with stage IIIA lung adenocarcinoma who achieved a partial response (PR) and 27.8 months of PFS after pembrolizumab therapy. The sum of ^18^F-FLT uptake (TLP) of the lesions at baseline decreased by 46.1% at 2 weeks after therapy, while the sum of the diameter was not changed (−0.1%). (B) A 76-year-old man with stage IIIC lung squamous cell carcinoma who achieved PR after proliferative pseudoprogression and 6.4 months of PFS after pembrolizumab therapy. ^18^F-FLT uptake of the primary lesion in the left lower lobe at baseline increased by 58.4% at 2 weeks after therapy followed by a decrease of ^18^F-FLT uptake (−31.4%) in 6-week PET images. (C) A 77-year-old man with stage IVA non-small cell lung cancer, not otherwise specified, who experienced PR followed by early acquired resistance and short PFS (5.2 months). TLP of the primary lesion in the left upper lobe at baseline dramatically decreased by 94.4% at 2 weeks after pembrolizumab therapy. However, the TLP at 2 weeks increased by 37.3% at 6 weeks after therapy. (D) A 71-year-old man with stage IVb lung adenocarcinoma who showed a dissociated response and experienced progressive disease and short PFS (3.3 months). TLP of the primary lesion of right S6 decreased by 100% at 2 weeks after therapy, while that of right hilar lymph node increased by 86.1%. ^18^F-FLT, 3′-deoxy-3′-[^18^F]-fluorothymidine; PET, positron emission tomography; PFS, progression-free survival; TLP, total lesion proliferation.

Figure 4 shows representative clinical images before and after anti-PD-1 therapy. Among eight responders (CR or PR), three patterns of ^18^F-FLT change were observed. First, an early proliferative response (ΔTLP0-2 <−30%) was observed in four (50%) of eight responders by ^18^F-FLT PET at 2 weeks after treatment initiation (figure 4A and online supplemental figure 2). The second pattern was a ‘proliferative pseudoprogression’ (figure 4B and online supplemental figure 3). Three of eight patients who were responders had increased TLP from baseline to 2 weeks (ΔTLP0-2 157.2%, 57.1%, and 37.4%, respectively; figure 2C), while all patients who responded showed decreased TLP from baseline to 6 weeks (figure 2F). All three patients were misdiagnosed as having progressive metabolic disease (PMD) using a ΔTLP0-2 cut-off value of 30% according to PERCIST.^25^ The third pattern was an ‘early proliferative relapse’. One patient who was a responder showed a rapid decrease of ^18^F-FLT accumulation at 2 weeks after treatment initiation followed by a relapsed finding on 6-week ^18^F-FLT PET, and there was a recurrence at 12 weeks after treatment initiation (figure 4C).

A few patients had some lesions that had an increased ^18^F-FLT uptake and others that decreased after treatment initiation, which is called a ‘dissociated response’. We defined the dissociated response as a concomitant relative TLP decrease >30% in some tumor lesions and a relative increase of >30% in others according to a previous report.^26^ Five (20%), among all patients, showed a dissociated response. Among them, one patient with PD as assessed by irRECIST was misdiagnosed as non-PD by ΔTLP0-6 with a 50% cut-off value (figure 4D).

## Discussion

This is the first prospective study to investigate the usefulness of ^18^F-FLT PET as an early predictive biomarker for the response to anti-PD-1 therapy in patients with advanced NSCLC. To our knowledge, there are three reports regarding immunotherapy response assessment with ^18^F-FLT PET in patients with melanoma^27–29^ and one report in those with prostate cancer.^30^ However, the sample sizes of these studies were small (n=5–17), and thus, the predictive capability and optimal timing of repeated ^18^F-FLT PET in these situations are not well elucidated. The present study showed that the change in ^18^F-FLT accumulation between baseline and 2 weeks of treatment (ΔTLP0-2) had a moderate predictive value for objective responses as assessed by irRECIST, and the changes between baseline and 6 weeks after treatment initiation (ΔSUV_max_0-6, ΔPTV0-6, and ΔTLP0-6) were able to predict responses more precisely as compared with ΔTLP0-2. Response assessments using ^18^F-FLT PET parameters may be able to assess treatment efficacy earlier than CT-based irRECIST. Furthermore, both ΔTLP0-2 and ΔTLP0-6 were significant prognostic factors for PFS, whereas known biomarkers such as tumor PD-L1 expression, ECOG-PS, and age were not.

Several previous studies have demonstrated the efficacy of ^18^F-FDG PET for predicting the response to immunotherapy in patients with advanced NSCLC. In many of these studies, serial PET scans were planned at approximately 2 months after treatment initiation.^31 32^ However, since some phase III trials using anti-PD-1 antibody in patients with NSCLC have shown that the median time to response is 8–9 weeks,^4–6^ the clinical benefits of the first response evaluation using PET scans at 2 months may be limited. In a recent study with a short-term evaluation, Kaira *et al*^33^ showed that the metabolic response by ^18^F-FDG uptake at 1 month after nivolumab treatment successfully predicted objective responses and survival. Furthermore, we recently reported the relationship between the change of ^18^F-FDG uptake at 2 weeks after nivolumab treatment and objective responses in patients with previously treated NSCLC.^15^ However, in this study, three of the nine responders exhibited an increased ^18^F-FDG uptake (>20%) between baseline and at 2 weeks, and thus ^18^F-FDG accumulation in tumor-infiltrating immune cells may cause an inflammatory metabolic pseudoprogression in early-phase PET imaging after anti-PD-1 therapy. Since ^18^F-FLT accumulation is considered to be a marker of cell proliferation, we expected that the predictive value of the changes in ^18^F-FLT PET parameters after 2 weeks would be superior to that of ^18^F-FDG PET parameters. However, the predictive accuracy of a ^18^F-FLT PET parameter (ΔTLP0-2) to predict PD as assessed by irRECIST in the current study was similar to that of the ^18^F-FDG PET parameter in our previous study (76.0% vs 76.0%, respectively),^15^ although the patient populations of those studies were not the same. However, the changes in tumor ^18^F-FLT uptake at 6 weeks after therapy showed a high accuracy of 90.9% for the prediction of PD and a significant correlation with PFS. Similarly, our previous study found that patients without relapse-associated findings on ^18^F-FDG PET at 8 weeks had significantly longer PFS than patients with PMD.^15^ Although a simple comparison of these two studies is impossible, serial ^18^F-FLT PET might be able to predict tumor response as quickly as or earlier than ^18^F-FDG PET. However, needless to say, ^18^F-FDG PET is more widely used than ^18^F-FLT PET, and there is more evidence for its effectiveness for tumor response assessment.

Among the response patterns we observed, three of eight patients who achieved PR showed increased TLP from baseline to 2 weeks. Of note, this discrepancy of response was not observed in 6-week ^18^F-FLT PET imaging. To our knowledge, this is the first report demonstrating the phenomenon of ‘proliferative’ pseudoprogression with ^18^F-FLT PET imaging. In addition to proliferative tumors, physiological ^18^F-FLT uptake has been observed in the bone marrow because of the high proliferative activity of hematopoietic cells in the marrow.^23 34^ Furthermore, ^18^F-FLT PET can also detect proliferative changes in spleens and lymph nodes after immunotherapy.^27–30^ Therefore, ^18^F-FLT uptake may be a reliable marker to assess the proliferative activity of immune cells. A recent study described the early pathological changes after neoadjuvant nivolumab therapy in patients with NSCLC.^14^ In that report, primary tumors with a major pathological response showed large numbers of infiltrating CD8+ T cells and macrophages. Meanwhile, an increase in the proliferation of CD8+ T cells in the blood within 4 weeks of anti-PD-1 therapy was observed in patients with advanced NSCLC, and it suggested an association with clinical benefit for that therapy.^35^ Thus, PD-1 blockade may induce the proliferation of CD8+ T cells in responding tumors and cause proliferative pseudoprogression in ^18^F-FLT PET imaging.

In the present study, one of five patients with dissociated response had an incorrect prediction for early response to immunotherapy on repeated ^18^F-FLT PET imaging. It has been reported that 7.7%–17.7% of NSCLC patients who are treated with immunotherapy experience dissociated responses in CT imaging.^36 37^ Some recent studies have indicated possible biological explanations for dissociated responses. First, there are specific differences in the immune tumor microenvironment at different metastatic sites,^38^ and these differences, including tumor-infiltrating lymphocyte status, can influence the response to immunotherapy.^39^ Second, since recent evidence suggests that multiple cytokines such as interferon-γ, IL-1α, and IL-27 induce or enhance PD-L1 expression in tumor cells,^40^ and the expression of these cytokines is influenced by the tumor microenvironment, PD-L1 expression can be heterogeneous among metastatic sites.^36 37^ Although dissociated responses in ^18^F-FLT PET imaging may lead to incorrect predictions of early response to immunotherapy, it can be advantageous to examine the changes in the proliferation of each lesion. Recently, new PET tracers, such as ^89^Zr-C4 and ^18^F-BMS-986192, that visualize tumor PD-L1 expression have been developed and show good correlation with tumor PD-L1 expression as measured by IHC and responses to immunotherapy.^41 42^ Since these PET images can reveal the heterogeneity of PD-L1 expression between different tumor lesions, they may predict dissociated responses prior to treatment initiation.

We found that ΔTLP was a better predictor for efficacy of anti-PD-1 therapy than ΔSUV_max_, which is in line with some previous reports using PET imaging for the response assessment of immunotherapy.^13 15 31^ Since it has been reported that PD-L1 expression is highly heterogeneous within tumors,^36^ the heterogeneity of tumor PD-L1 expression may reflect heterogeneous infiltration of inflammatory cells and cause a dissociated result between SUV_max_ and TLP in response to anti-PD-1 therapy. Thus, volumetric analysis (PTV or TLP) of PET imaging may be a more appropriate method for the response assessment to immunotherapy as compared with SUV_max_.

This study has some limitations. ^18^F-FLT PET is limited when assessing liver and bone metastases because of the high background uptake in bone marrow and liver. Another limitation is the small sample size, so that the reliability of the cut-off value of this study is not certain. Furthermore, as described in the Materials and methods section, we examined various thresholds and decided that an SUV of 2.0 was most suitable for volumetric analysis of ^18^F-FLT PET in patients with NSCLC, though full validation of this SUV threshold is needed in the future. However, the present initial observation is valuable for future studies to establish appropriate timing of follow-up ^18^F-FLT PET scanning, a cut-off value for response assessment, and an SUV threshold for volumetric analysis. Further studies with larger samples are required to examine the efficacy of ^18^F-FLT PET in predicting the early response to immunotherapy.

## Conclusion

The results of this study suggest that ^18^F-FLT PET is a useful tool for predicting early response to anti-PD-1 therapy in patients with NSCLC. The changes in ^18^F-FLT uptake as early as 2 weeks after treatment initiation had a moderate predictive value for subsequent treatment efficacy, although proliferative pseudoprogression was observed in 37.5% of responders. However, the changes between baseline and 6 weeks after treatment initiation were robust biomarkers of treatment efficacy. Further studies with larger sample sizes would be beneficial to validate this conclusion.

## Data Availability

Data are available on reasonable request.
