# Supplementary material for: Predictive value of 3′-deoxy-3′-18F-fluorothymidine PET in the early response to anti-programmed death-1 therapy in patients with advanced non-small cell lung cancer
Source: J Immunother Cancer. 2021 Jul 21;9(7):e003079. doi: 10.1136/jitc-2021-003079 (PMC8296775; doi:10.1136/jitc-2021-003079)
Supplement: Supplementary data [file jitc-2021-003079supp001.pdf]

Fig. S1. ROC curve analysis of ^{18}F-FLT PET parameters.

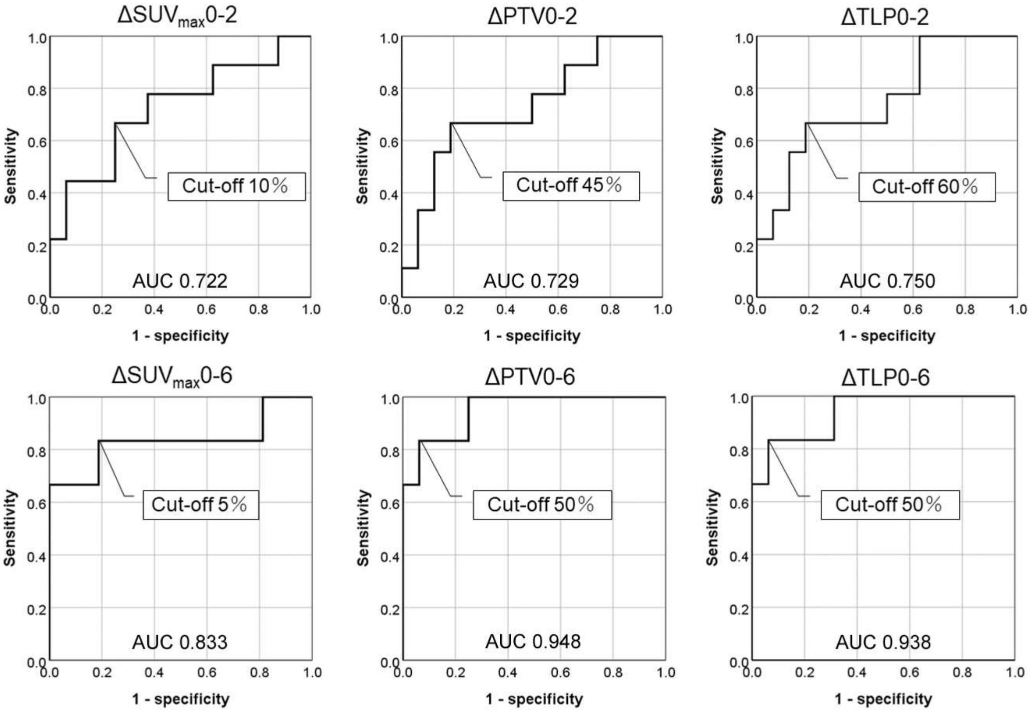

Curves were constructed to determine the ability of ΔSUV_{max}, ΔPTV, and ΔTLP to predict disease progression after anti-PD-1 therapy. The optimal cut-off values for ΔSUV_{max}0-2, ΔPTV0-2, ΔTLP0-2, ΔSUV_{max}0-6, ΔPTV0-6, and ΔTLP0-6 were estimated to be 5%, 45%, 60%, 5%, 50%, and 50%, respectively.

SUV, standardized uptake value; PTV, proliferative tumor volume; TLP, total lesion proliferation; AUC, area under curve

Fig. S2. Representative PET and CT images with a 67-year-old woman with stage IIIC lung squamous cell carcinoma in her right lower lobe who achieved a partial response (PR) after pembrolizumab therapy.

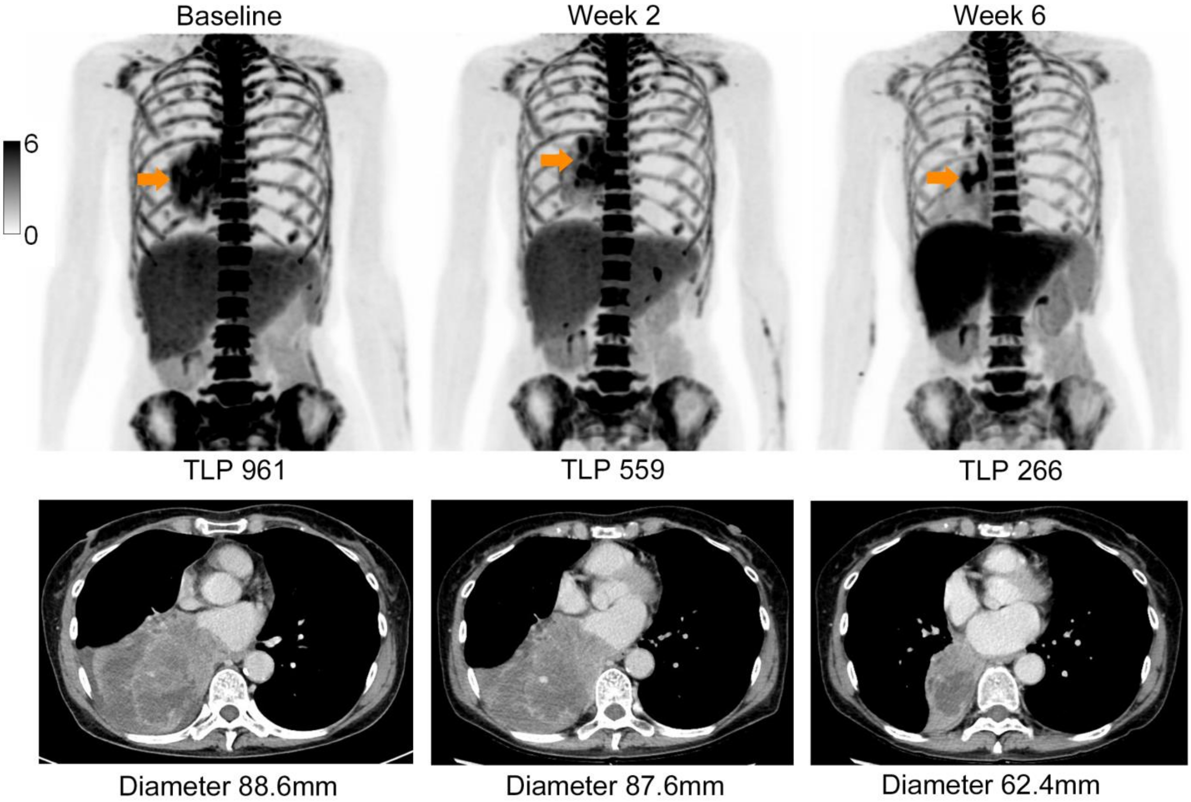

The sum of ^{18}F-FLT uptake (TLP) of the lesions at baseline decreased by 41.8% at 2 weeks after therapy (arrow). Chest CT at 2 weeks after therapy showed the sum of diameter of the lesions was not changed (-0.8%), while the sum of diameter at 6 weeks after therapy decreased by 29.6% from baseline.

TLP, total lesion proliferation

Fig. S3. Representative PET and CT images with a 68-year-old man with stage IVA lung squamous cell carcinoma in the right lower lobe who achieved SD and long PFS (7.0 months) after nivolumab therapy.

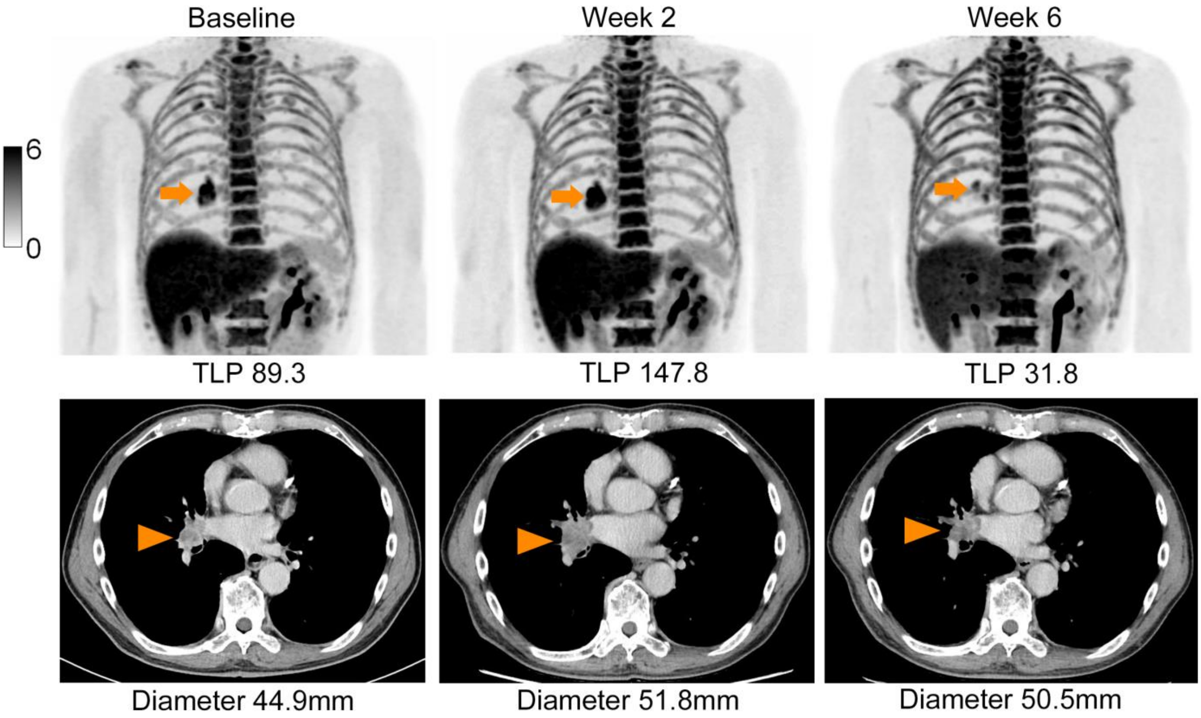

^{18}F-FLT uptake (TLP) of the right hilar lymph node (arrow) at baseline increased by 65.5% at 2 weeks after therapy followed by a decrease of ^{18}F-FLT uptake (-64.3%) in 6-week PET images. Chest CT images showed the diameter of the right hilar lymph node (arrowhead) at baseline increased by 15.4% at 2 weeks after therapy.

TLP, total lesion proliferation

Sato M, *et al. J Immunother Cancer* 2021; 9:e003079. doi: 10.1136/jitc-2021-003079
